# Brain Volumetric Alterations in Preclinical HIV-Associated Neurocognitive Disorder Using Automatic Brain Quantification and Segmentation Tool

**DOI:** 10.3389/fnins.2021.713760

**Published:** 2021-08-11

**Authors:** Ruili Li, Yu Qi, Lin Shi, Wei Wang, Aidong Zhang, Yishan Luo, Wing Kit Kung, Zengxin Jiao, Guangxue Liu, Hongjun Li, Longjiang Zhang

**Affiliations:** ^1^Department of Medical Imaging, Jinling Hospital, Medical School of Nanjing University, Nanjing, China; ^2^Department of Radiology, Beijing Youan Hospital, Capital Medical University, Beijing, China; ^3^BrainNow Research Institute, Shenzhen, China; ^4^Department of Imaging and Interventional Radiology, The Chinese University of Hong Kong, Hong Kong, Hong Kong; ^5^Department of Natural Medicines, School of Pharmaceutical Sciences, Peking University Health Science Center, Beijing, China

**Keywords:** HIV, neurocognitive disorder, brain volumetric alterations, MR imaging, T_1_-weighted imaging

## Abstract

**Purpose:**

This study aimed to determine if people living with HIV (PLWH) in preclinical human immunodeficiency virus (HIV)-associated neurocognitive disorder (HAND), with no clinical symptoms and without decreased daily functioning, suffer from brain volumetric alterations and its patterns.

**Method:**

Fifty-nine male PLWH at the HAND preclinical stage were evaluated, including 19 subjects with asymptomatic neurocognitive impairment (ANI), 17 subjects with cognitive abnormality that does not reach ANI (Not reach ANI), and 23 subjects with cognitive integrity. Moreover, 23 healthy volunteers were set as the seronegative normal controls (NCs). These individuals underwent sagittal three-dimensional T_1_-weighted imaging (3D T_1_WI). Quantified data and volumetric measures of brain structures were automatically segmented and extracted using AccuBrain^®^. In addition, the multiple linear regression analysis was performed to analyze the relationship of volumes of brain structures and clinical variables in preclinical HAND, and the correlations of the brain volume parameters with different cognitive function states were assessed by Pearson’s correlation analysis.

**Results:**

The significant difference was shown in the relative volumes of the ventricular system, bilateral lateral ventricle, thalamus, caudate, and left parietal lobe gray matter between the preclinical HAND and NCs. Furthermore, the relative volumes of the bilateral thalamus in preclinical HAND were negatively correlated with attention/working memory (left: *r* = −0.271, *p* = 0.042; right: *r* = −0.273, *p* = 0.040). Higher age was associated with increased relative volumes of the bilateral lateral ventricle and ventricular system and reduced relative volumes of the left thalamus and parietal lobe gray matter. The lower CD4^+^/CD8^+^ ratio was associated with increased relative volumes of the left lateral ventricle and ventricular system. Longer disease course was associated with increased relative volumes of the bilateral thalamus. No significant difference was found among preclinical HAND subgroups in all indices, and the difference between the individual groups (Not reach ANI and Cognitive integrity groups) and NCs was also insignificant. However, there was a significant difference between ANI and NCs in the relative volumes of the bilateral caudate and lateral ventricle.

**Conclusion:**

Male PLWH at the HAND preclinical stage suffer from brain volumetric alterations. AccuBrain^®^ provides potential value in evaluating HIV-related neurocognitive dysfunction.

## Introduction

**FIGURE 1:**
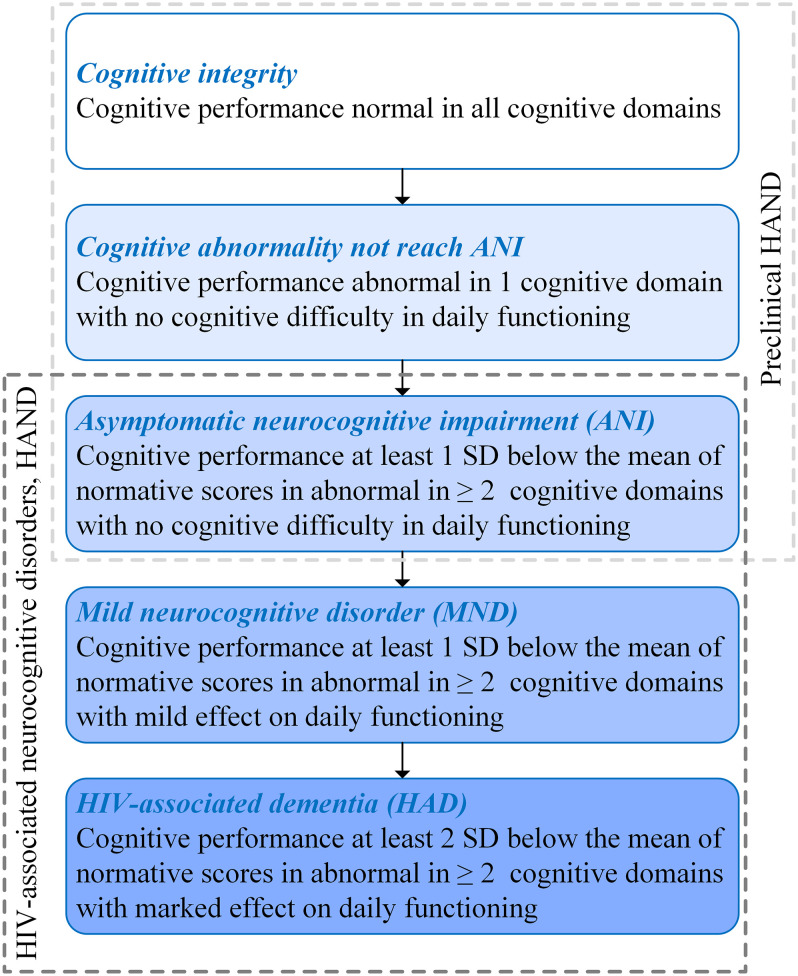
Subclassification of human immunodeficiency virus (HIV)-associated neurocognitive disorder (HAND) and preclinical HAND (Antinori et al., 2007).

Human immunodeficiency virus (HIV) can penetrate the central nervous system (CNS) after seroconversion at the initial stage of infection, subsequently leading to varying degrees of cognitive impairment, which is defined as HIV-associated neurocognitive disorder (HAND) (Antinori et al., 2007). Depending on the extent of cognitive impairment, HAND can be divided into three types: HIV-associated dementia (HAD), which represents a markable interference with daily functioning; mild neurocognitive disorder (MND) for describing a mild effect on functioning; and asymptomatic neurocognitive impairment (ANI) for describing a clinically asymptomatic. Moreover, ANI was taken into account by performance at least 1 SD below the mean of normative scores in ≥2 cognitive domains with no cognitive difficulties in everyday functioning (Antinori et al., 2007) (shown in Figure 1).

The HIV-associated mortality and morbidity can be controlled effectively by using combined antiretroviral therapy (cART), which has successfully transformed HIV/AIDS from a high-fatality disease into a controllable chronic disease. Moreover, the progression of HIV-related cognitive impairment has also been changed, which are manifested as the fact that HAND is characterized by HAD in the pre-cART era, while ANI is the majority in HAND in the post-cART era (Simioni et al., 2010). Despite reducing the degree of cognitive impairment, HAND is still a significant public health concern, because approximately half of people living with HIV (PLWH) have milder forms of HAND (Wright et al., 2015), which may cause decreased compliance of cART, lower ability to perform complex daily tasks, and decline in quality of life and employment difficulties (Letendre et al., 2010). Moreover, in the post-cART era, some PLWH present cognitive impairment in only one domain. For these patients, their cognitive function has abnormalities that do not reach the level of ANI. Prior studies demonstrated that HIV-related brain injury is covertly active or progressing in the majority of virus-suppressed individuals (Cysique et al., 2018), and the pathophysiological changes occurred before the changes in neuropsychological manifestations (Garvey et al., 2014). Therefore, PLWH with cognitively intact may already have neuropathological abnormalities. In this study, PLWH with ANI, cognitive abnormalities that do not reach ANI (Not reach ANI), or cognitive intact was considered as preclinical HAND (shown in Figure 1). It was known that the neurocognitive impairment of PLWH gradually aggravates over time and still has the risk of dementia and death (Cysique et al., 2018). The prevalence and impact of the preclinical HAND on PLWH gain importance gradually, and several guidelines have suggested screening all PLWH for HAND (Mind Exchange Working Group, 2013; British HIV Association, 2018; European AIDS Clinical Society, 2019).

Of note, magnetic resonance (MR) neuroimaging techniques are common non-invasive methods and could have a great application value in the diagnosis, treatment, and pathological research of HAND. Among these techniques, structural magnetic resonance imaging (MRI) based on three-dimensional T_1_-weighted imaging (3D T_1_WI) can objectively and clearly show brain atrophy, which may help to understand the neuropathological changes of HAND. Regarding the potential pathological mechanism of HIV-associated brain damage, in the pre-cART era, significant volume reductions have been robustly demonstrated in the cerebral gray matter assessed by 3D T_1_WI, especially in the subcortical regions (basal ganglia) and posterior cortex (Aylward et al., 1993, 1995; Woods et al., 2009), and the caudate nucleus showed progressive atrophy related to the disease stage and the rate of decline in CD4^+^ cell counts (Stout et al., 1998). More recent neuroimaging studies conducted in the post-cART era have demonstrated that the atrophy occurs not only in the subcortical regions but also in the cortical areas, and in the expansion of the ventricle (Thompson et al., 2005; Ances et al., 2012; Ragin et al., 2012). At present, there is still a lack of consistent understanding of the changing trend of brain volume in PLWH with slightly cognitive function impairment, and the characteristics of the change remain poorly described. It has been reported that gray matter volume reductions spread diffusely across the whole brain in PLWH diagnosed as ANI or non-HAND (Kato et al., 2020). In contrast, in another study, no statistically significant difference in brain volumes between non-HAND and normal controls (NCs) was found (Heaps et al., 2015).

Efforts to prevent the disease process after the onset of neurocognitive symptoms have not been achieved in recent years. Thus, it is best to start the trial at the preclinical stage of the disease before symptoms appear. The innovation of our study is to focus on the preclinical stage of HAND and to subgroup the preclinical HAND according to the degree of cognitive impairment, to capture the dynamic changes of brain structure in preclinical stage. Indeed, the research on brain microstructure change of different cognitive states of preclinical HAND has several prognostic benefits: (1) it may inform whether neuroimaging can detect brain micro-changes in preclinical HAND patients earlier, even in patients with intact cognition; (2) it may assist in understanding the micro-change patterns of brain tissue volume in preclinical HAND patients with different cognitive states from the neuroimaging view; and (3) the profile of changes in early HIV-related brain injury can also provide unique vision into the fundamental neuropathogenic processes. Nowadays, there were no studies that have investigated the quantitative brain volumetric changes pattern of different cognitive states in preclinical HAND.

In recent years, quantitative analysis, automatic brain segmentation, and machine learning have been widely studied in various neurodegenerative diseases (Wenzel et al., 2018; Dou et al., 2020; Ansart et al., 2021). However, many classification methods based on machine learning are highly dependent on limited training data and lack basic clinical evidence (Bron et al., 2017). In addition, these methods are regarded as a “black box” and difficult for clinicians and radiologists without computer expertise to make out how and why these artificial intelligence techniques make a decision, so the lack of interpretation limits their extensive application in daily clinical practice (Kelly et al., 2019). The ideal imaging-aided diagnosis method should be objective, easy to understand and operate, and economical and efficient. AccuBrain^®^ has been clinically applicable with Conformitè Européenne (CE) and Food and Drug Administration (FDA) approval and is considered to be a research-validated fully automatic neuroanatomical volumetric segmentation tool that can efficiently quantify various brain structure volumes in about 20 min, including brain parenchyma, ventricular regions, and atrophy of lobar regions (Mai et al., 2021; Yu et al., 2021). AccuBrain^®^ has the ability to avoid variability induced by the difference in software parameter settings and could provide an incomparable user experience with respect to ease of use, which also enables a smooth translation of the research findings to clinical practice (Liu et al., 2020). The superiority of AccuBrain^®^ in brain structure segmentation accuracy and reproducibility was also shown in another research (Abrigo et al., 2019). There may be only slight brain structure differences in preclinical HAND patients with different cognitive states; a segmentation tool with higher accuracy and reproducibility was necessary, such as AccuBrain^®^.

The objective of this study was to investigate if preclinical HAND patients with no clinical symptoms and no decline in daily function suffer from brain volumetric alterations and change patterns by using a fully automatic brain quantification and segmentation tool. Furthermore, the possible relationship between cognitive/clinical variables and brain volume micro-changes has been also examined.

## Materials and Methods

### Subjects

This study was approved by the Institutional Review Boards of Beijing YouAn Hospital, Capital Medical University. Written informed consent was acquired from all subjects after a detailed description of this study. All PLWH were enrolled from the infectious disease clinic of Beijing YouAn Hospital, Capital Medical University. Study inclusion criteria for patients included ≥18 years of age, male, and HAND preclinical stage. The exclusion criteria for participants included (1) long-term alcohol or drug abuse (more than 6 months); (2) a current or past neurological and psychiatric disorders: stroke, trauma, tumors, infection (except HIV), multiple sclerosis, epilepsy, anxiety, and depression; (3) MND and HAD, two other serious forms of HAND; and (4) contraindication to MRI. Sixty-one PLWH were recruited, among which 23 were normal cognitive function, 18 cognitive abnormalities do not reach ANI, and 20 ANI. For control purposes, 23 seronegative healthy volunteers matched to PLWH by age, gender, and educational level were enrolled from the same community as the infected subjects via advertisements. The NC participants were excluded if they had obvious brain lesions or any history of previous neurological disease.

The disease course was determined based on the patients’ self-reports of their risk behaviors. The history of cART was identified from medical records. Clinical laboratory examinations (plasma HIV RNA levels, recent plasma CD4^+^ cell count, and CD4^+^/CD8^+^ ratio) were measured within 2 weeks of the MRI examination. The neurocognitive evaluation was performed within 3 h before MR scan. Age, gender, educational level, disease course, laboratory examinations, and neuropsychological performance status were available and listed in Table 1.

**TABLE 1:** Baseline characteristics of study subjects.

	ANI (*n* = 19)	Not reach ANI (*n* = 17)	Cognitive integrity (*n* = 23)	Normal control (*n* = 23)	*p*-Value
Age (years)	28.0(24.0, 31.0)	32.65 ± 8.95	32.22 ± 6.91	32.09 ± 4.46	0.218^*a*^
Education (years)	16.0(16.0, 18.0)	16.0(16.0, 16.0)	16.0(16.0, 16.0)	16.0 (16.0, 16.0)	0.138^*a*^
Disease course (month)	20.0(5.0, 33.0)	27.88 ± 18.39	24.0(9.0, 43.0)	Not available	0.725^*a*^
CD4^+^	537.39 ± 228.93	530.37 ± 138.74	522.77 ± 226.60	Not available	0.974^*b*^
CD4^+^/CD8^+^	0.52(0.41, 0.68)	0.63(0.41, 0.80)	0.54 ± 0.23	Not available	0.528^*a*^
Virologically controlled (%)	63.2%(12/19)	70.6% (12/17)	73.9%(17/23)	Not available	0.748^*c*^
cART (%)	73.7%(14/19)	82.4%(14/17)	82.63%(19/23)	Not available	0.862^*d*^

*ANI, asymptomatic neurocognitive impairment; Not reach ANI, cognitive abnormality not reach ANI; Virologically controlled, viral load in plasma below the detection limit of the instrument.*

*^*a*^Kruskal–Wallis test.*

*^*b*^One-way analysis of variance (ANOVA).*

*^*c*^Chi-square test.*

*^*d*^Fisher’s exact test; significance level *p* < 0.05.*

### Neuropsychological Tests

At 2.5 h before MR scan, each patient underwent a battery of neuropsychological assessments, containing six ability domains and a report of functional difficulties in daily life. Self-questionnaires of daily functional status were evaluated with a short Activities of Daily Living scale (Gandhi et al., 2011). The neurocognitive status surveys the following abilities: (1) verbal fluency using Animal Verbal Fluency Test (AFT); (2) attention/working memory using Continuous Performance Test-Identical Pair (CPT-IP), Wechsler Memory Scale (WMS-III), and Paced Auditory Serial Addition Test (PASAT); (3) executive function using Wisconsin Card Sorting Tests (WCST-64); (4) memory (learning and delayed recall) using Hopkins Verbal Learning Test (HVLT-R) and Brief Visuospatial Memory Test (BVMT-R); (5) speed of information processing using Trail-Making Test A (TMT-A); and (6) fine motor skills using Grooved Pegboard (dominant and non-dominant hands) (Heaton et al., 2010). The raw score for every test was transformed into T-scores, adjusted for age, sex, and years of education. Attention/working memory included three cognitive tests; and memory (learning and delayed recall) included two cognitive tests. Averaged T-score of multiple tests was computed for that cognitive domain. In case of two or more cognitive domain impairment (performance of at least 1 SD below the normative scores) with no cognitive difficulties in daily life, ANI was considered. In PLWH whose cognitive impairment involved one cognitive domain and presented no everyday functioning difficulties, cognitive abnormality Not reach ANI should be taken into account. Twenty-three subjects in this study were diagnosed as Cognitive integrity, 18 patients were classified as Not reach ANI, and 20 patients were considered as ANI according to the Frascati criteria (Antinori et al., 2007).

### Magnetic Resonance Imaging Data Acquisition

Siemens 3.0T MRI Scanner (Tim Trio, Siemens, Erlangen, Germany) was used to acquire images. A 32-channel phased-array head coil with tight but comfortable foam pads was equipped to minimize head motion. Earplugs were used to reduce scanner noise. T_2_-weighted fluid-attenuated inversion recovery (T_2_-FLAIR) combined fat saturation [repetition time (TR) = 8,000 ms, echo time (TE) = 2,370.9 ms, and inversion time (TI) = 97 ms] sequence was acquired to check whether there was a visible intracranial lesion. Subsequently, a high-resolution, three-dimensional, T_1_-weighted, magnetization-prepared rapid gradient echo scan was acquired: TR = 1,900 ms, TE = 2.52 ms, TI = 900 ms, field of view = 250 × 250 mm, acquisition matrix = 256 × 246, number of slices = 176, slice thickness = 1 mm, flip angle = 9°, and voxel size = 1 × 0.977 × 0.977 mm^3^. The scan duration was 4 min 18 s.

### Image Processing

The obtained T_1_-weighted scans were first visually evaluated to confirm that there were no serious common artifacts (such as motion or metal artifacts) and no obvious brain atrophy or lesions, which may lead to inaccurate volume estimation from the images. One case with Not reach ANI and one ANI case were excluded due to poor image quality with severe motion artifacts. Thus, imaging data of 59 patients (23 cases of Cognitive integrity, 17 cases of Not reach ANI, and 19 cases of ANI) and 23 NCs were presented for analysis.

AccuBrain^®^ (BrainNow Medical Technology Limited, Shenzhen, China) is a fully automatic brain quantification and segmentation tool. AccuBrain^®^ has been demonstrated to have a superior reproducibility against the difference in scanner selection and scanning protocols. Comparing the data acquired by three clinical scanners with different brands and default parameter settings, the inter-scanner reproducibility of the quantification results of AccuBrain^®^ is lower than that of FreeSurfer v6.0 and FSL v5.0 (Liu et al., 2020). In another study that validates the hippocampus segmentation results, the mean Dice Similarity Coefficient of the segmentation results generated from AccuBrain^®^ is higher than that of FreeSurfer by around 20% (Abrigo et al., 2019). The compatibility of AccuBrain^®^ under different acquisition conditions was also demonstrated. Different from other research software, AccuBrain^®^ is a fully automatic cloud-based computational tool, with no need for parameter settings during use (Abrigo et al., 2019). More technical characteristics and their comparison with other automatic brain image segmentation methods were described in detail in previous work (Gao et al., 2020; Wang et al., 2020). This tool has been validated to be more accurate, objective, and easier to implement and has been widely used in various neurological and mental diseases (Wang et al., 2019; Zhao et al., 2019; Dou et al., 2020; Mai et al., 2021; Yu et al., 2021).

**FIGURE 2:**
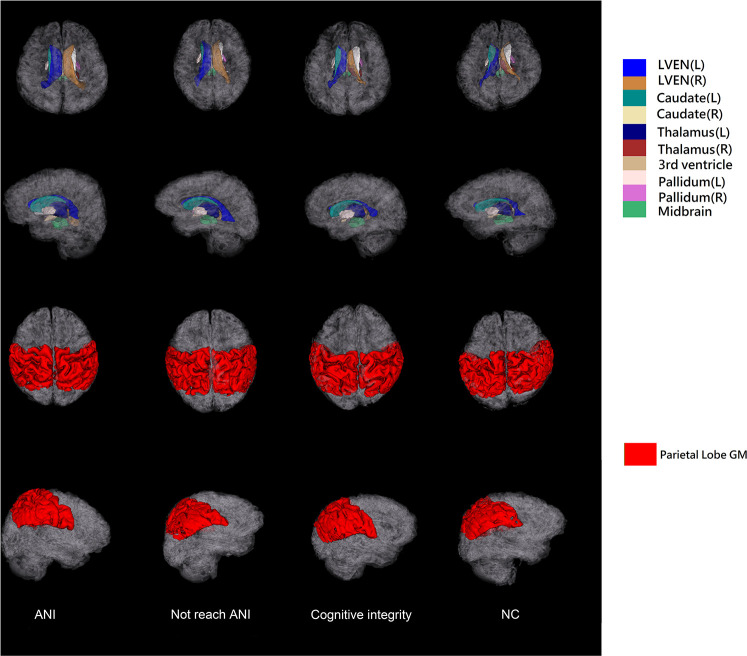
The illustration of 3D surfaces of brain structure segmentations of the four groups. The first two rows show the 3D surfaces of selected brain structures with the cortical surfaces displayed in semi-transparent gray color. The last two rows show the surfaces of parietal lobe gray matter. L, left; R, right; GM, gray matter; ANI, asymptomatic neurocognitive impairment; Not reach ANI, cognitive abnormality but do not reach the level of ANI; NC, normal control.

The T_1_-weighted MRI data were all processed using AccuBrain^®^ v1.2 to extract quantified data and volumetric measures of brain structures. It automatically segments the three major brain tissues (gray matter, white matter, and cerebrospinal fluid) and other brain structures (thalamus, hippocampus, ventricular system, etc.) with high accuracy (Abrigo et al., 2019). AccuBrain^®^ v1.2 used a multi-atlas-based image registration scheme (Luo, 2018). The atlas pool contains a large number of brain MR images obtained from different scanners, together with their anatomical labels. According to the similarities between the atlas images and the image to be segmented, several atlases were selected from the atlas pool, and then non-rigid image registrations were performed to project the selected atlases into the individual space. The labels in individual space were fused to form the final segmentation result. Sixty-two anatomical labels, including different brain substructures and cortical regions, were obtained from the segmentation result. The volumetric information includes the absolute volume and relative volume (normalized by intracranial volume) of different brain structures. Absolute volume is the actual volume of each brain region (in ml). To correct for the skull size difference, the relative volume was extracted as our volumetric brain measures for analysis. The relative volume of each brain region was defined as the absolute volume divided by the individual’s total intracranial volume (Yu et al., 2021). The brain structures and lobe segmentations obtained from example data in each group for a visual inspection are shown in Figure 2.

In addition to brain structure volumetric, AccuBrain^®^ can also calculate lobar atrophy indices of the cortical regions, including the left and right parts of the frontal lobe, occipital lobe, temporal lobe, parietal lobe, cingulate lobe, and insular lobe. The lobar atrophy index quantifies the atrophy degree by calculating the ratio of cerebrospinal fluid volume to brain parenchyma volume (gray matter and white matter) in the specific lobe region (Luo, 2018). As well as lobar atrophy index, the relative gray matter volume in each lobe can also be calculated.

### Statistical Analyses

The normality of all variables was analyzed by the Shapiro–Wilk test, and any *p*-value above 0.05 indicates normality. The *p*-values of the Shapiro–Wilk test are shown in Supplementary Table 1. Furthermore, non-normality variables are shown in Table 1 as median (interquartile range), and normality variables were reported as mean ± SD. For non-normality variables, the Kruskal–Wallis test (non-parametric test) was employed to compare the age, education, disease course, and CD4^+^/CD8^+^ ratio in different groups. Analysis of variance (ANOVA; parametric test) was performed to compare the CD4^+^ values (normality) in different groups. For categorical variables, Fisher’s exact test or chi-square test was used. All information about the statistical methods is listed in the notes of Table 1. The multivariate linear regression analysis was performed to assess the association between the relative volumes of brain structures and clinical variables. The dependent variable was the volumes of brain structures that showed significant differences between preclinical HAND and NCs. The independent variables were age, disease course CD4^+^, and CD4^+^/CD8^+^. Pearson’s correlation analysis was employed to assess the relationship between the relative volumes of brain structures and cognitive tests in the preclinical HAND group. All analyses were performed with IBM SPSS Statistics for Windows, version 22.0 (IBM Corp., Armonk, NY, United States). All tests are two-tailed, and the *p*-values < 0.05 were considered statistically significant.

To investigate the difference among the four study groups (ANI, Not reach ANI, Cognitive integrity, and NCs), an independent-samples *t*-test with false discovery rate (FDR) correction was performed on 62 quantities, including 38 brain structure relative volumes and 24 lobar indices calculated by AccuBrain^®^. The FDR correction performed on the *t*-test results follows the method mentioned by Benjamini and Hochberg (1995). The *p*-values of the 62 quantities were listed in order, from the smallest to the largest. Then each *p*-value was compared with its Benjamini–Hochberg critical value, (*i*/*n*) × *Q*, where *i* is the rank of the quantity, *n* is the total number of quantities, and *Q* is the FDR (0.05 in this case). The largest *p*-value that is smaller than its critical value and all *p*-values smaller than their critical values were considered as significant. First, the ANI, Not reach ANI, and Cognitive integrity were combined to the preclinical HAND group and compared with NCs, and then each of the four groups was compared with each other.

## Results

### Study Population

Demographics and clinical characteristics of participants are listed in Table 1. Fifty-nine participants met the criteria for preclinical HAND, including 19 ANI, 17 Not reach ANI, and 23 Cognitive integrity. There were no significant differences in age, gender, or educational level among the three HIV-positive groups and NCs. There were no significant differences in the disease course, CD4^+^, CD4^+^/CD8^+^, rate of virologically controlled, and rate of cART among the three HIV-positive groups.

### Brain Volumetry Findings

**FIGURE 3:**
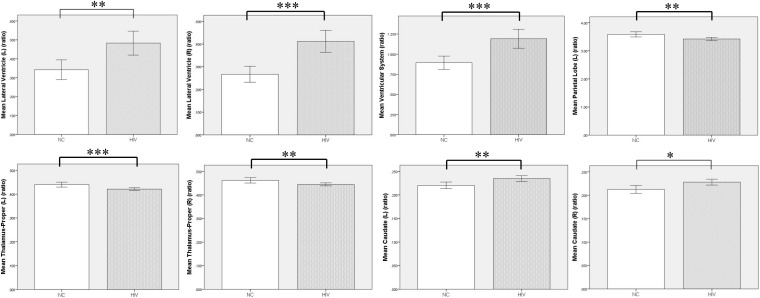
Volumetric comparison between preclinical HAND group and normal control (NC) group. Compared with the NC group, the HIV patients at the HAND preclinical stage showed significant enlargement in ventricular system, bilateral lateral ventricle, and caudate, and significant atrophy in bilateral thalamus and left parietal lobe gray matter. L, left; R, right; NC, normal control; HAND, HIV-associated neurocognitive disorder. Error bars show 95%CI; *p < 0.05; **p < 0.01; and ***p < 0.001.

Compared with the NC group, the preclinical HAND group (grouping ANI, Not reach ANI, and Cognitive integrity) showed significant atrophy in the bilateral thalamus; and the left parietal lobe gray matter, the ventricular system, and bilateral lateral ventricle were significantly enlarged (*p* < 0.05). Notably, the enlargement of the bilateral caudate was observed rather than atrophy (*p* < 0.05). The relative volume differences between preclinical the HAND and NC groups in the *t*-test with FDR correction are displayed in Table 2 and Figure 3.

**TABLE 2:** The brain structures that showed significant relative volume differences between preclinical HIV-associated neurocognitive disorder (HAND) and normal control (NC) groups in *t*-test with false discovery rate (FDR) correction.

	Preclinical HAND		NC			
	Mean*	Std	Mean*	Std	*t* value	*p*-Value
Lateral ventricle (L)	0.4811	0.2474	0.3471	0.1308	3.173	0.01
Lateral ventricle (R)	0.4083	0.1872	0.2713	0.0872	4.504	<0.001
Ventricular system	1.1847	0.4560	0.9048	0.2040	3.832	<0.001
Parietal lobe GM (L)	3.4232	0.2372	3.6152	0.2096	–3.587	0.009
Thalamus (L)	0.4217	0.0205	0.4377	0.0247	–2.755	<0.001
Thalamus (R)	0.4459	0.0247	0.46	0.0276	–2.185	0.0052
Caudate (L)	0.2345	0.0231	0.2185	0.0164	3.501	0.009
Caudate (R)	0.2278	0.0245	0.212	0.0204	2.969	0.03

*The negative *t* value means that preclinical HAND has a smaller mean relative volume than the NC group. Significance level *p* < 0.05.*

L, left; R, right; HAND, HIV-associated neurocognitive disorder; FDR, false discovery rate; NC, normal control; GM, gray matter.

*The unit of the ratio is 100%.

**FIGURE 4:**
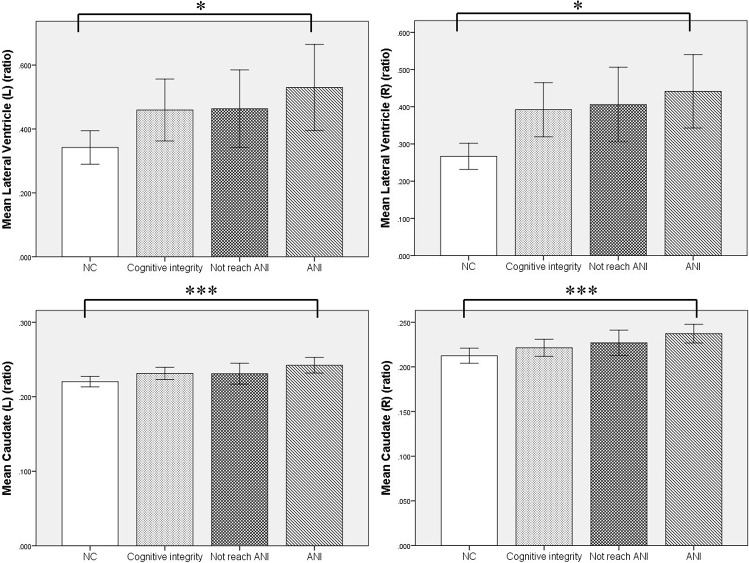
The relationship of the relative volumes of brain structures among the four groups. Compared with the NC group, the asymptomatic neurocognitive impairment (ANI) group showed significant enlargement in the relative volume of bilateral lateral ventricle and caudate. Although there was no significant difference in other in-groups, the relative volume of bilateral lateral ventricle and caudate showed a trend of gradual increase, in the following order: NC (normal control), Cognitive integrity, Not reach ANI (cognitive abnormality but do not reach the level of ANI), and ANI (asymptomatic neurocognitive impairment). L, left; R, right. Error bars show 95%CI; *p < 0.05 and ***p < 0.001.

Furthermore, MRI volumetric and atrophic pattern differences among all groups included in the study (ANI, Not reach ANI, Cognitive integrity, and NCs) were investigated to catch the brain microstructure change process of different cognitive states in preclinical HAND. However, significant differences were found only between the ANI and NC groups. Compared with NCs, the ANI group showed significant enlargement in the relative volume of the bilateral lateral ventricle; and also unexpectedly, in the bilateral caudate, there is an enlargement rather than atrophy (*p* < 0.05). Although there was no significant difference found among HIV-positive groups (ANI, Not reach ANI, and Cognitive integrity) and between HIV-positive groups and NCs in all indices, the relative volume of the bilateral lateral ventricle and caudate showed a trend of gradual increase in order, from NCs to Cognitive integrity, Not reach ANI, and ANI (shown in Table 3 and Figure 4).

**TABLE 3:** The brain structures that showed significant relative volume differences between asymptomatic neurocognitive impairment (ANI) and NC groups in *t*-test with FDR correction.

	ANI		NC			
	Mean*	Std	Mean*	Std	*t* value	*p*-Value
Lateral ventricle (L)	0.5298	0.2794	0.3471	0.1308	2.622	0.049
Lateral ventricle (R)	0.4414	0.2049	0.2713	0.0872	3.376	0.048
Caudate (L)	0.2422	0.0219	0.2185	0.0164	3.901	<0.001
Caudate (R)	0.2373	0.0216	0.212	0.0204	3.865	<0.001

*The positive *t* value means that ANI has a smaller mean relative volume than the NC group. Significance level *p* < 0.05.*

L, left; R, right; ANI, asymptomatic neurocognitive impairment; NC, normal control; FDR, false discovery rate.

*The unit of the ratio is 100%.

### The Association Between the Volumes of Brain Structures and Clinical Variables, and Cognitive Tests

**FIGURE 5:**
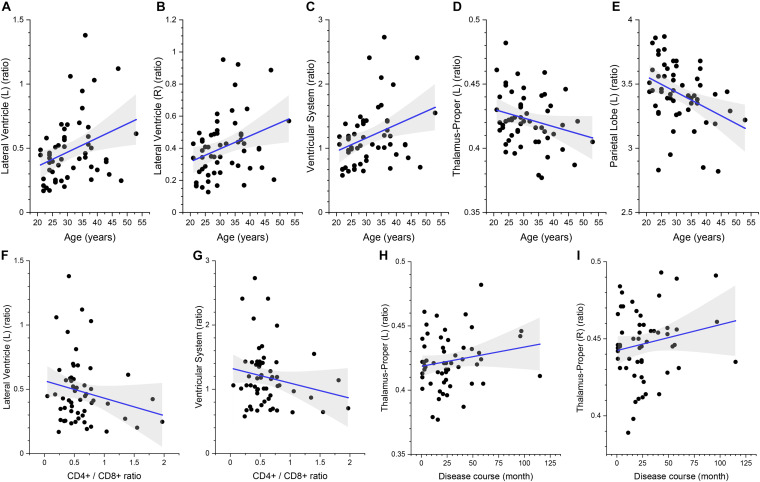
The association between the relative volumes of brain structures and clinical variables. Higher gray matter volume in the bilateral lateral ventricle **(A,B)**, ventricular system **(C)**, lower volume in the left thalamus **(D)**, and parietal lobe gray matter **(E)** are associated with higher age. Higher gray matter volume in the left lateral ventricle **(F)** and ventricular system **(G)** is associated with lower CD4^+^/CD8^+^ ratio. Higher gray matter volume in the bilateral thalamus **(H,I)** is associated with longer disease course. The shaded area shows its 95% confidence interval.

The multivariate linear regression analysis between clinical variables and brain volume of preclinical HAND patients showed that higher age was associated with increased relative volumes of the lateral ventricle (L) [β = 0.427 (95%CI, 0.151–0.703), *p* = 0.003], lateral ventricle (R) [β = 0.419 (95%CI, 0.138–0.699), *p* = 0.004], and ventricular system [β = 0.426 (95%CI, 0.149–0.703), *p* = 0.003] and reduced relative volumes of the thalamus (L) [β = −0.352 (95%CI, −0.637 to −0.067), *p* = 0.017] and parietal lobe (L) [β = −0.292 (95%CI, −0.571 to −0.014), *p* = 0.04] (shown in Table 4 and Figures 5A–E). The lower CD4^+^/CD8^+^ ratio was associated with increased relative volumes of the lateral ventricle (L) [β = −0.327 (95%CI, −0.613 to −0.041), *p* = 0.026] and ventricular system [β = −0.315 (95%CI, −0.602 to −0.028), *p* = 0.032] (shown in Table 4 and Figures 5F,G). Longer disease course was associated with increased relative volumes of the thalamus (L) [β = 0.326 (95%CI, 0.045–0.607), *p* = 0.024] and thalamus (R) [β = 0.308 (95%CI, 0.022–0.594), *p* = 0.035] (shown in Table 4 and Figures 5H,I).

**TABLE 4:** Effects of HIV-associated clinical variables on brain volume of preclinical HAND patients.

Dependent variable	Independent variable	Unstandardized coefficients		Standardized coefficients		*p*-Value
		β	95%CI	β	95%CI	
Lateral ventricle (L) (ratio)	Age	0.014	[0.005, 0.024]	0.427	[0.151, 0.703]	0.003**
	Disease course	−0.001	[−0.003, 0.002]	–0.053	[−0.325, 0.220]	0.699
	CD4^+^	7.334E-5	[<0.001, <0.001]	0.059	[−0.218, 0.337]	0.668
	CD4^+^/CD8^+^	−0.219	[−0.410, −0.027]	–0.327	[−0.613, −0.041]	0.026*
Lateral ventricle (R) (ratio)	Age	0.010	[0.003, 0.017]	0.419	[0.138, 0.699]	0.004**
	Disease course	−0.001	[−0.003, 0.002]	–0.067	[−0.343, 0.210]	0.631
	CD4^+^	2.494E-5	[<0.001, <0.001]	0.027	[−0.254, 0.308]	0.847
	CD4^+^/CD8^+^	−0.139	[−0.283, 0.006]	–0.279	[−0.569, 0.011]	0.059
Ventricular system (ratio)	Age	0.026	[0.009, 0.043]	0.426	[0.149, 0.703]	0.003**
	Disease course	−0.001	[−0.006, 0.004]	–0.036	[−0.309, 0.236]	0.790
	CD4^+^	<0.001	[−0.001, 0.001]	0.052	[−0.226, 0.329]	0.710
	CD4^+^/CD8^+^	−0.387	[−0.740, −0.035]	–0.315	[−0.602, −0.028]	0.032*
Thalamus – proper (L) (ratio)	Age	−0.001	[−0.002, <0.001]	–0.352	[−0.637, −0.067]	0.017*
	Disease course	<0.001	[<0.001, 0.001]	0.326	[0.045, 0.607]	0.024*
	CD4^+^	−5.489E-6	[<0.001, <0.001]	–0.053	[−0.339, 0.233]	0.711
	CD4^+^/CD8^+^	−0.003	[−0.019, 0.014]	–0.045	[−0.340, 0.250]	0.761
Thalamus – proper (R) (ratio)	Age	−0.001	[−0.002,<0.001]	–0.274	[−0.564, 0.016]	0.064
	Disease course	<0.001	[<0.001, 0.001]	0.308	[0.022, 0.594]	0.035*
	CD4^+^	−2.882E-7	[<0.001, <0.001]	–0.003	[−0.293, 0.288]	0.986
	CD4^+^/CD8^+^	−0.007	[−0.026, 0.011]	–0.119	[−0.420, 0.181]	0.429
Caudate (L) (ratio)	Age	<0.001	[−0.001, 0.001]	–0.047	[−0.355, 0.262]	0.763
	Disease course	7.323E-5	[<0.001, <0.001]	0.077	[−0.227, 0.381]	0.615
	CD4^+^	3.080E-6	[<0.001, <0.001]	0.027	[−0.283, 0.336]	0.864
	CD4^+^/CD8^+^	−0.001	[−0.021, 0.019]	–0.018	[−0.337, 0.302]	0.911
Caudate (R) (ratio)	Age	−3.040E-5	[−0.001, 0.001]	–0.009	[−0.315, 0.298]	0.954
	Disease course	8.152E-5	[<0.001, <0.001]	0.079	[−0.224, 0.381]	0.603
	CD4^+^	5.290E-6	[<0.001, <0.001]	0.042	[−0.265, 0.350]	0.785
	CD4^+^/CD8^+^	−0.009	[−0.031, 0.012]	–0.135	[−0.452, 0.183]	0.398
Parietal lobe (L) (ratio)	Age	−0.009	[−0.018, <0.001]	–0.292	[−0.571, −0.014]	0.040*
	Disease course	−0.002	[−0.005, 0.001]	–0.208	[−0.483, 0.067]	0.135
	CD4^+^	−6.863E-5	[<0.001, <0.001]	–0.058	[−0.338, 0.221]	0.677
	CD4^+^/CD8^+^	−0.005	[−0.189, 0.179]	–0.008	[−0.297, 0.280]	0.954

L, left; R, right; HAND, HIV-associated neurocognitive disorder.

*p < 0.05; **p < 0.01.

**FIGURE 6:**
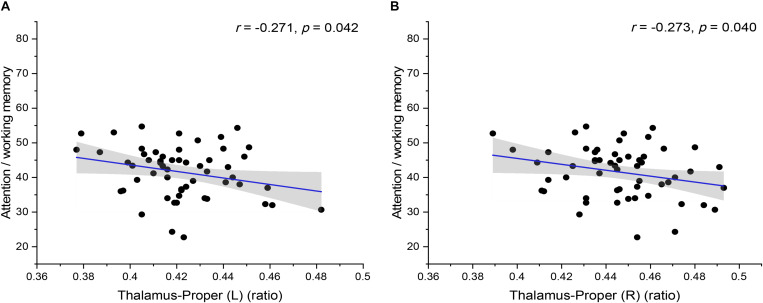
The correlation between the relative volumes of brain structures and cognitive variables. The relative volumes of bilateral thalamus-proper [**(A)** left and **(B)** right] were negatively correlated with attention/working memory scores. The shaded area shows its 95% confidence interval. L, left; R, right. Significance level *p* < 0.05.

In addition, the statistical analysis of correlations between brain volume and cognitive tests of preclinical HAND patients showed that there was a significant negative correlation between brain volume and cognitive tests of preclinical HAND patients found, including the thalamus (L) and attention/working memory (*r* = −0.271, *p* = 0.042), thalamus (R), and attention/working memory (*r* = −0.273, *p* = 0.040) (Figure 6). In the initial statistical analysis, notably, there was one outlier in the scatter plot of disease course and one outlier in the scatter plot of attention/working memory score. All statistical analyses were conducted after excluding outliers.

## Discussion

In the post-cART era, preclinical HAND, including Cognitive integrity and ANI (the mildest subtype of HAND), was much more frequent than MND and HAD (Benjamini and Hochberg, 1995). Although milder, ANI can still affect the cognitive function of PLWH and may progress to MND or HAD, so it is necessary to clarify the early diagnosis and neuropathogenesis of preclinical HAND. To the best of our knowledge, the brain microstructure change characteristics of different cognitive states in preclinical HAND have not been described, which play an important role in evaluating disease progression, clarifying pathological mechanisms, and implementing possible interventions. Automated brain volumetry was used in this research to assess the alterations of brain microstructure volumes in PLWH with preclinical HAND (including ANI, Not reach ANI, and Cognitive integrity groups). The results showed that compared with the NC group, in the preclinical HAND group, the bilateral caudate, lateral ventricle, and ventricular system were enlarged, and the bilateral thalamus and left parietal lobe gray matter were atrophied. Furthermore, there was a negative correlation between the bilateral thalamus and attention/working memory in preclinical HAND. Age, CD4^+^/CD8^+^, and disease course were significantly associated with altered brain volumes. Then, the results of cognitive sub-stratification showed that there was no significant difference in all indices among the three HIV groups. No difference in the relative volume of each brain region was observed between the individual groups (Not reach ANI and Cognitive integrity groups) and NC group. However, compared with NCs, enlargement of the bilateral caudate and lateral ventricle was found in the ANI group.

The results of this research showed the enlargement of the ventricular system and bilateral lateral ventricles in the preclinical HAND group compared with NCs, which is consistent with previous studies (Jernigan et al., 1993). Furthermore, to capture the process of the lateral ventricles volume change, the preclinical HAND group was subgrouped according to the degree of cognitive dysfunction. The relative volume of the bilateral lateral ventricle showed a trend of gradual increase when compared among three HIV subgroups (Cognitive integrity, Not reach ANI, and ANI groups). This observation suggested that the periventricular white matter may be gradually destroyed and resulted in gradual loss of cognitive function after HIV infection. The periventricular white matter such as the corpus callosum and central white matter is vulnerable to viral attack (Peluso et al., 2013; Ragin et al., 2015; Li et al., 2018). The virus in the cerebrospinal fluid could penetrate the cerebrospinal fluid brain barrier and directly or indirectly cause damage around the lateral ventricles, which is a possible explanation. White matter damage and disconnection from the cortex may occur in PLWH with milder cognitive impairment, and the pathological changes are likely to be the axonal chronic injury (Li et al., 2018; Zhao et al., 2020) and may cause the atrophy of the gray matter, and then the bilateral lateral ventricles expand. Furthermore, in this research, the lower CD4^+^/CD8^+^ ratio was associated with larger left lateral ventricle and ventricular system. As far as we know, the lower CD4^+^/CD8^+^ ratio could be attributed to the persistent inflammation and immunosenescence caused by viral infection (Sainz et al., 2013) and can also be used as a biomarker for T-cell activation to characterize the migration of T cells into the CNS after HIV infection and the production of inflammatory cytokines, which can indirectly lead to white matter damage. Our study also found that age was positively correlated to the relative volumes of the bilateral lateral ventricle and ventricular system and negatively correlated to relative volumes of the left thalamus and parietal lobe gray matter. According to the previous research, aging is an important factor that could affect HAND development and progression (Becker et al., 2009). One reason was that the brain degenerated with age, and the volume of both the cortex and white matter reduced. Another reason may be that there is an interaction between aging and HIV infection, and HIV infection can accelerate the atrophy rate of the cortical and subcortical regions (Sanford et al., 2018a,b) compared with those in the healthy control. The mechanism of interaction may include the increase of oxidative stress and proinflammatory mediators (Rumbaugh and Tyor, 2015).

Moreover, the bilateral thalamus showed atrophy in preclinical HAND compared with NCs, which is consistent with previous research findings in that the atrophy was also observed in the same region even in the neurocognitively asymptomatic stage (Tesic et al., 2018). For early-infected HIV individuals, the decreased volume in the thalamus may be related to hypometabolism, and hypometabolism in the thalamus was more pronounced than in the whole brain in HIV participants compared with NCs (Hammoud et al., 2018). The thalamus mainly consists of cortical projection thalamic cortical neurons and is considered as a main subcortical component. Thalamic nuclei play a role in attentional processing by controlling sensory signals and controls interactions within and across cortical regions, and the thalamic injury could potentially be related to functional abnormalities (Wimmer et al., 2015; Zhou et al., 2016; Halassa and Kastner, 2017; Schmitt and Halassa, 2017). In this research, the bilateral thalamus volumes of PLWH in preclinical HAND were negatively correlated with attention/working memory, which verified the previous speculation that changes in thalamic volume could lead to dysfunction though only in attention/working memory. We also found that the disease course was positively correlated with bilateral thalamus volumes. Although in the long term, it could be expected that the brain volume would shrink with the longer disease course. Immune activation is a possible explanation for the increase in bilateral thalamus volume with the duration of infection in the early preclinical stage of HAND. The atrophy of the left parietal lobe gray matter was also shown in the preclinical HAND group compared with NCs. A study found that HIV can be isolated from the parietal lobe at autopsy of a patient who had been infected with HIV for 15 days, which suggested that HIV has a greater impact on this area in the early stage of infection (Hamdeh et al., 2015). In our study, only the left parietal lobe gray matter showed atrophy in all cerebral cortices; this may also manifest that the parietal lobe is more vulnerable to HIV attacks than any other parts of the cerebral cortex.

The change of relative volume of the bilateral caudate was a controversial research point. A study reported the enlargement of the bilateral caudate after HIV infection (Castelo et al., 2007), while the atrophy was found in other researches on patients who had a longer duration of infection (Ances et al., 2012; Egbert et al., 2018). In our study, the relative volume of the bilateral caudate enlarged in preclinical HAND compared with NCs. To clarify enlargement or atrophy of the bilateral caudate in PLWH, the subgroup of our study was further analyzed, and the results showed that the relative volume of the bilateral caudate in ANI was still larger than that in NCs. However, when comparing among three HIV subgroups (Cognitive integrity, Not reach ANI, and ANI groups), there was no significant difference. The relative volumes of the caudate showed an increasing trend. Alakkas et al. (2019) also found no significant difference between ANI and neurocognitively unimpaired in the volume of any gray matter, but they neither used HIV-negative healthy individuals as a control group to compare with ANI nor grouped preclinical HAND, so it failed to capture the trend of volumetric change. To further explain the changing pattern of the relative volumetric of the caudate in early-HIV infection and the neuropathological mechanism, three hypotheses were proposed, as follows. First, metabolism in basal ganglia is increased in the early stages of infection, and decreased metabolism was found later in the disease process (Rottenberg et al., 1996; Hammoud et al., 2018). All PLWH in our research were in the stage of preclinical HAND, a stage in which the basal ganglia metabolism is vigorous. HIV viral proteins can trigger inflammatory activity, which is considered to be the neuropathogenesis of neurocognitive impairment in HIV. Metabolic and inflammatory influences may lead to an increase in the relative volume of the caudate. Second, the enlargement of the caudate may be caused by the reorganization of brain structure and function. The thalamus contains non-specific thalamic nuclei including the central nucleus, which receives fibers from the cerebellum, brainstem, and other thalamic nuclei and then projects them into the striatum, including the caudate. The reduction in thalamic volume suggests that there may be some functional reduction; this may result in a functional and volumetric compensatory increase in the caudate. Furthermore, increased activation in the caudate during a phonemic fluency task has been found in HIV individuals (Thames et al., 2016). Our previous study found that early-infected HIV individuals had an increased amplitude of low-frequency fluctuation (ALFF) in the caudate regardless of cART (Li et al., 2019). The abnormal function activation and increased ALFF may also lead to a volumetric temporary increase in the caudate. Lastly, due to the smaller number of samples in subgroup analysis, this may lead to statistical bias. A larger sample size will help to clarify the changing pattern of relative volumetric of the caudate.

According to the diagnostic criteria of HAND, Not reach ANI only involves one cognitive domain, does not belong to HAND, and is defined as preclinical HAND. The results of this research showed that patients in the stage of Not reach ANI exhibited a trend of volumetric atrophy, but the MRI volumetric difference between Not reach ANI and NC groups is not significant. The results demonstrated that the Frascati diagnostic criteria of HAND are sensitive and can identify more early preclinical HAND patients than MRI neuroimaging methods, or the micro-changes of brain volume in the Not reach ANI group have not been observed due to the sensitivity of the observation methods in this study. In the future, we will try to employ other methods to study the brain structure and function alterations of the Not reach ANI group.

Although milder, the neurocognitive function of PLWH with Not reach ANI and ANI stage is still impaired and may progress to MND or HAD as HIV+ individuals age (Heaton et al., 2012). Early detection and diagnosis are crucial to the treatment of preclinical HAND. During the past decade, there has been an increasing interest in the mechanism, diagnosis, and treatment of HAND. However, the effective treatment of HAND has not been established, which indicates that the pathogenesis underlying the HAND remains controversial and unclear (Irollo et al., 2021). In the absence of updated neuropathology research, much of the knowledge on HAND will have to rely on advanced neuroimaging technology, which can provide insights on macrolevel brain changes. The results of this research indicate that AccuBrain^®^ can identify preclinical HAND and can capture the dynamic change patterns of brain volume alterations in different stages of preclinical HAND, which is shown as a gradual volumetric alteration process. Recent researches suggested that more subtle neuronal changes might drive the pathology of HAND in cART era, such as synaptic injury (Gelman, 2015; Ru and Tang, 2017). Such alterations in brain volume may suggest synaptic damage and contribute to neurocognitive impairment. However, the neuroimaging method of this research cannot detect micro-changes in small structural components of neurons, such as the synapses and dendritic spines. Further studies are necessary to validate whether the abnormalities described in our study are related to the pathogenesis of this condition. Previous research reported that reversing synaptic cartilage damage in animal models can restore cognitive function, so it highlights a promising treatment approach that has the potential to treat HAND (Irollo et al., 2021). Moreover, the studies on the manifestations and mechanisms of synaptic damage and functional and structural network abnormity in preclinical HAND patients and experimental models may contribute to developing safe and effective therapeutic methods that reverse subtle neuropathological changes and cognitive impairment, while longitudinal research might assess the risk factors for developing HAND and the response to treatment.

There are several limitations to this study. First, only single-center data and a small sample set were involved, especially for the number of cohorts in subgroup analysis, which may lower the statistical power; multicenter and expanding datasets are needed in the future. Second, all subjects were male, which may prevent other populations from benefiting from this research, especially female PLWH. Third, the cross-sectional design of the present research limits the ability to catch the more real dynamic process of brain volume changes; longitudinal studies on preclinical HAND are necessary. Fourth, only the volumetric indicators from T_1_ structural MRI are analyzed in this study; the combination of multi-MRI parameters will be helpful, such as structure connection, network, and resting-state functional markers.

In summary, our study indicated that in the stage of preclinical HAND, male PLWH showed an increased relative volume in the bilateral caudate and lateral ventricles, and decreased relative volume in the bilateral thalamus and left parietal lobe gray matter. The bilateral thalamus can reflect attention/working memory function; and the age, CD4^+^/CD8^+^ ratio, and disease course could be associated with brain volume, which revealed the structural underpinnings of brain dysfunction. Another finding was that when comparing the subgroups of preclinical HAND (Cognitive integrity, Not reach ANI, and ANI groups), the relative volumes of the bilateral lateral ventricle and the caudate showed a trend of gradual increases; this may indicate that there are certain characteristic changes in the brain tissue during the stage of preclinical HAND. AccuBrain^®^ provides potential value in evaluating early HIV-related neurocognitive dysfunction.

## Data Availability Statement

The raw data supporting the conclusions of this article will be made available by the authors, without undue reservation.

## Ethics Statement

The studies involving human participants were reviewed and approved by the Institutional Review Boards of Beijing Youan Hospital, Capital Medical University. The patients/participants provided their written informed consent to participate in this study.

## Author Contributions

RL, YQ, LS, GL, HL, and LZ were involved in the design and conduct of this study. RL, YQ, LS, WW, AZ, YL, and WK contributed to the data analysis and the result interpretation. RL, YQ, LS, and GL wrote the first draft of the manuscript. WW, AZ, YL, WK, ZJ, HL, and LZ wrote portions of the manuscript and reviewed the whole manuscript. All authors have read and approved the manuscript.

## Conflict of Interest

The authors declare that the research was conducted in the absence of any commercial or financial relationships that could be construed as a potential conflict of interest.

## Publisher’s Note

All claims expressed in this article are solely those of the authors and do not necessarily represent those of their affiliated organizations, or those of the publisher, the editors and the reviewers. Any product that may be evaluated in this article, or claim that may be made by its manufacturer, is not guaranteed or endorsed by the publisher.
