# Supplementary material for: Brain Volumetric Alterations in Preclinical HIV-Associated Neurocognitive Disorder Using Automatic Brain Quantification and Segmentation Tool
Source: Front Neurosci. 2021 Aug 11;15:713760. doi: 10.3389/fnins.2021.713760 (PMC8385127; doi:10.3389/fnins.2021.713760)
Supplement: Supplementary file 1 [file Table_1.DOCX]

Table S1. The P values of Shapiro-Wilk test for the normality of continuous variables

|  | ANI  (n = 19) | Not reach ANI  (n = 17) | Cognitive integrity  (n = 23) | Normal control  (n = 23) |
| --- | --- | --- | --- | --- |
| Age (years) | 0.013^*^ | 0.223 | 0.131 | 0.427 |
| Education (years) | <0.001^*^ | <0.001^*^ | <0.001^*^ | <0.001^*^ |
| Disease course (month) | <0.001^*^ | 0.457 | 0.001^*^ | Not available. |
| CD4^+^ | 0.706 | 0.898 | 0.239 | Not available. |
| CD4^+^/CD8^+^ | <0.001^*^ | 0.008^*^ | 0.123 | Not available. |

Note: ANI, asymptomatic neurocognitive impairment; Not reach ANI, cognitive abnormality not reach ANI; ^*^Significance level P < 0.05.
